# Being ‘The Only Consumer’ – Exploring the Lived Experiences of Health Consumer Representatives in Australian Health System Involvement

**DOI:** 10.1111/hex.70901

**Published:** 2026-10-05

**Authors:** Hannah Fielder, Rebecca Kelly, Phoebe Griffin

**Affiliations:** ^1^ University of Tasmania, Tasmanian School of Medicine Hobart Tasmania Australia; ^2^ The George Institute for Global Health, University of New South Wales Sydney New South Wales Australia; ^3^ University of Tasmania, Tasmanian School of Medicine Launceston Tasmania Australia

**Keywords:** consumers, engagement, health system, involvement, patient and public involvement

## Abstract

**Introduction:**

Health consumer representatives (HCRs) are increasingly expected to contribute to decision‐making across the health system, including at the level of policy, strategy and system‐wide governance. However, while HCR involvement is relatively well established at the direct‐care and health service‐level context, system level involvement remains underdeveloped and poorly understood as a distinct form of participation. In addition, and arguably because of this, HCR involvement at this level continues to face the persistent challenges, such as role confusion and tokenistic ‘tick‐a‐box’ engagement, despite increasing policy commitments. This study explores the lived experiences of HCRs in Australia to better understand how system‐level involvement is experienced in practice.

**Methods:**

Thirteen HCRs from across Australia were purposively sampled and participated in semi‐structured one‐to‐one interviews conducted via Zoom or telephone. Interviews were audio‐recorded, transcribed and analysed iteratively using an interpretive phenomenological approach.

**Results:**

Participants’ experiences were captured by the prominent theme of **being the ‘only consumer’**, reflecting both the uniqueness of their role and expertise, but also the feeling of marginalisation they face. Two cross‐cutting influences shaped this experience: **power dynamics**, including structural and interpersonal inequities and **relational factors**, particularly allyship and supportive relationships. One key element of the consumer experience was the process of **becoming an expert** as they gained knowledge of systems and confidence in the value of the consumer perspective. Another was **finding meaning** in their work both through everyday interactions and long‐term contributions to systems change.

**Conclusion:**

The quality of HCR involvement in system‐level spaces is shaped not only by governance structures, organisational processes and policy commitments, but also by power dynamics, relationships and the extent to which lived experience is recognised as unique expertise. Underpinning this, the ability for HCRs to find meaning in their roles and in genuine partnerships is what moves their involvement beyond tokenism into supported and valued participation. These findings highlight that meaningful system‐level HCR involvement depends not only on having consumers ‘in the room’, but on recognising their expertise, sharing power, building respectful relationships, and designing structures that allow their contributions to influence decisions.

**Patient or Public Contribution:**

Investigator HF holds the dual role of researcher and HCR peak body worker and has personal lived experience of being an HCR at the health‐system level. The inception of the research question was grounded in this lived experience and informed by regular input from two highly experienced HCR peers. Study participants contributed to refining themes and the interpretation of data. Importantly, the central objective of the research was to elevate the insights, reflections, and lived experiences of HCRs.

## 1 Introduction

The World Health Organization's Alma‐Ata Declaration in 1978 stated that: ‘The people have a right and duty to participate individually and collectively in the planning and implementation of their health care.’ [1]. Since then, health legislators and accreditation bodies across the world have increasingly required their health systems and services to enable users of health systems to have influence over decision‐making [2]. In Australia, health consumer involvement is becoming an expectation [3], with consumers having a legislated right to partnership within healthcare organisations. These organisational changes towards consumer involvement have progressed because of and alongside the growth of the health consumer movement [4]. This movement, led by consumer champions globally and in Australia, continues to advocate for increased opportunities for involvement, alongside a stronger focus on ensuring it is meaningful [5].

Despite policy support, meaningful involvement is not consistently achieved in practice [6] and health consumer involvement can vary widely in its depth and influence, with tokenism persisting [4]. Frameworks like the International Association for Public Participation spectrum of public participation [7] describe a range of participation from ‘Inform’, through to ‘Empower’. While all levels may be appropriate depending on the context [8], involvement is generally considered meaningful when it involves genuine influence over decisions [9]. Tokenism also occurs when promised levels of influence are not realised in practice, or when health consumers have markedly less power than other stakeholders [10].

Health consumer involvement can and should take place at all levels of the health system [11]. These can be conceptualised as ranging from direct care‐level about individual health, to service‐level decisions about health service design, delivery and evaluation, through to health system‐level decisions about policy, strategy, accreditation and broader health system governance [12]. While the understanding and practice of health consumer representation is relatively well established at the direct‐care and service levels [8], system‐level involvement is more complex and less clearly defined. This is because consumers are removed from their role as patients and instead ‘collaborate as equal partners, contributing unique skills while sharing information and perspectives toward innovative ideas that contribute to the overall improvement of health care’ (13, p. 497).

At the service‐ and system‐level, involvement often occurs through individuals acting as health consumer representatives (HCRs) who participate in focus groups, workshops, committees and other decision‐making forums [13]. Terminology varies widely across contexts, with individuals known as a carer‐, family‐, health consumer‐ or lived experience‐ representative, advocate or expert. For this study the term health consumer representative (HCR) will be used to capture all these identities and experiences.

Over the past three decades, internationally and in Australia, hard‐fought changes in policy have strengthened the presence of HCRs within health systems [14]. Recent international literature reviews of ‘system‐level’ HCR involvement have investigated methods [8], impact [15, 16] and barriers and facilitators to best practice [17, 18, 19]. However, much of this literature focuses on service‐level involvement or incorporates the perspectives of health professionals alongside consumers. There remains a dearth of publications exploring the experience of system‐level involvement from the perspectives of HCRs themselves.

In Australia, while some recent studies explore the experience of HCR involvement [20, 21, 22, 23], there is little rich description of what it is like to be an HCR involved at a system level. A deeper exploration of these lived experiences is needed to inform more meaningful and effective partnerships. Therefore, this study aims to explore the experiences of HCRs involved in system‐level health decision‐making in Australia.

## 2 Methods and Framework

### 2.1 Research Design

This study draws on constructivist principles and an interpretative phenomenological (hermeneutic) approach to generate meaning from in‐depth interviews [24]. These focus on HCRs’ rich descriptions of their experiences in health‐system involvement. This approach assumes that descriptions of people's life worlds are a form of truth [25] and allows for meaning to be built from words people use to describe phenomena [26]. Further, hermeneutics proposes that the researcher must acknowledge, describe, and draw upon their own inextricable worldviews in the process of understanding and analysing the data [27]. The lead researcher has prior involvement supporting health consumer representatives and advocating for their inclusion in decision‐making through their professional work, which could become a bias. The hermeneutic approach instead enables these prior experiences to be used as a clearly described lens (see Section 2.5).

### 2.2 Participants

Participants from across Australia with HCR experiences at the health system‐level were recruited. For this study, health systems‐level involvement was defined as contributing to health systems change, health policy or health‐related public policy at the Australian State or National level. Eligible participants identified as HCRs and had current or recent (within the past 6 months) health systems‐level experience.

Participants were excluded if their involvement was primarily clinical, focused on a specific disease/condition or confined to a small geographic area. Additional exclusions were current or recent (within 12 months) health professionals or those regularly employed by a health‐related government department.

### 2.3 Recruitment

Purposive sampling [28] was used to ensure participants had specific insights relevant to the study aim. HCRs were recruited from a range of health system‐level decision‐making settings, including workshops, committees, boards, panels, forums and projects (hereafter referred to as ‘groups’). Potential participants were invited through social media pages, newsletters and other regular communication channels of health consumer organisations across Australia. Participants either contacted researcher HF directly via email to express interest or requested to be contacted via Microsoft Forms.

Suitability for inclusion was determined through a preliminary phone conversation with HF. If necessary, suitability was discussed between the research team and decided by consensus. In addition, recruitment purposively included participants with a range of different lived experiences, as well as HCR roles in a variety of health system‐level roles. Written consent to participate was sought after eligibility was determined and prior to the commencement of interviews.

### 2.4 Data Collection and Analysis

HF conducted one‐to‐one, semi‐structured interviews via Zoom or telephone between December 2024 and March 2025. Interviews lasted approximately 1 h and were conversational, allowing flexibility to elicit rich descriptions of experiences. Consistent with an interpretive phenomenological (hermeneutic) method [29] the interview schedule evolved iteratively, although key questions remained the same (Appendix 1). Interviews were recorded and transcribed using Otter. ai (v3.79.0). Transcripts were checked by two members of the research team and de‐identified by HF. After each interview HF wrote a reflection on the interview to document impressions, assumptions and potential influences of their insider position as a researcher with HCR experience.

Transcript data were imported into NVivo 15.1 and analysed using a hermeneutic approach [26]. This approach involved an iterative cycle of reading and re‐reading that allowed the researcher (HF) to make linkages between participant experiences and concepts and ideas. This led to the creation of preliminary themes, which informed further data collection through slight changes to the interview questions [24]. Draft themes were revised within the research team, who were familiar with the transcript data, during regular team meetings. A second researcher (PG) independently reviewed nine transcripts to check for theme development at the end of the analysis period.

### 2.5 Reflexivity and Trustworthiness

The hermeneutic method used proposes that researchers acknowledge, describe, and draw upon their own worldviews in the process of understanding and analysing the data [27]. As such HF's reflections formed a part of discussions as a reflexive tool to allow HF's insider lens to be acknowledged and examined. Research team discussion introduced an outsider perspective that, alongside HF's reflections, helped the researchers to identify and examine potential insider bias. Potential bias was considered and addressed during the development of the themes. To ensure fidelity, final draft themes were sent to participants for comment. Six participants responded with three providing feedback, which was considered alongside the data in the final stages of refining themes.

## 3 Results

Thirteen semi‐structured in‐depth interviews were undertaken with participants from Tasmania (*n* = 3), South Australia (*n* = 3), Queensland (*n* = 3), Victoria (*n* = 2), Western Australia (*n* = 1) and Australian Capital Territory (*n* = 1). Participants reported between less than two and 20 years of experience as HCRs in system‐level settings. Identities and attributes that participants used to describe themselves included being male, female, transgender, person of colour, LGBTIQ+ and/or neurodivergent. Two participants described themselves as relatively young (compared to others in the field), others as parents with children who have neurodivergence and/or significant physical or mental health conditions. Participants described themselves as consumer representatives, family and carer representatives, health/consumer advocates, patient safety advocates and activists.

### 3.1 Summary of Themes

Five interrelated group experiential themes describing the experience of HCRs in system‐level involvement were identified from the individual interviews (Figure 1).

**Figure 1:**
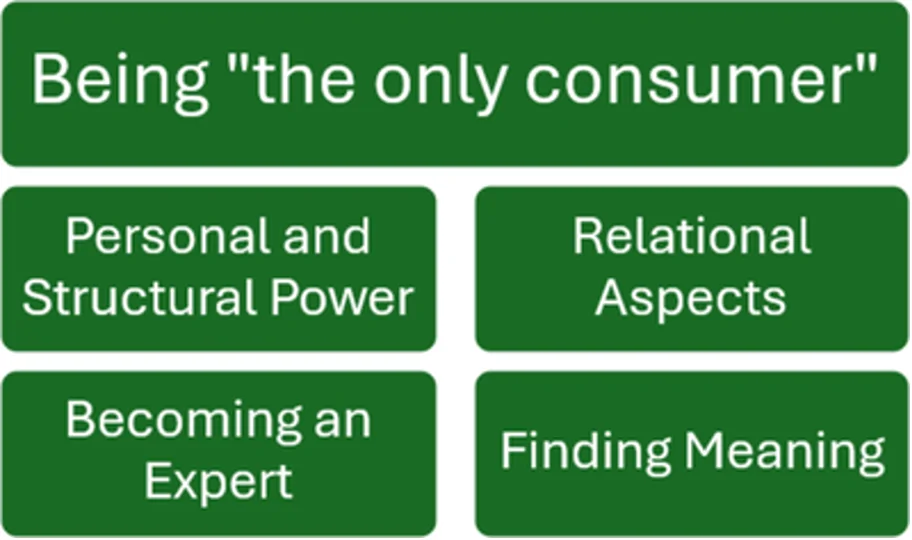
Overview of the relationship of themes relating to health consumer representative involvement in system‐level spaces. Interrelationship of themes: The prominent experience of being ‘the only consumer’ framed the way HCRs viewed their experiences. Effects of personal and structural power and relational aspects provided the context for HCR experiences. Becoming an expert and finding meaning were key elements that shaped participants’ HCR experiences.

### 3.2 Themes

#### 3.2.1 Theme 1 – Being ‘The Only Consumer’

The experience of being the ‘only consumer’ was a pervasive theme. It included being the only HCR ‘in the room’, sometimes feeling ostracised and having a significantly different perspective to others in the group. While this was not always experienced by all participants in all HCR opportunities, it was a common enough occurrence within and between individual stories to frame experiences as well as the phenomenon of HCR involvement more generally.

Participants often found themselves being the only person representing the HCR perspective. Sometimes, because of this, they felt like they were not part of a team:

[talking about being the only consumer on a committee] Sometimes that can be hell…where it's all clinicians, I don't feel part of the team. I feel that I am a consumer rep.

P6

Often this experience of being ‘othered’ could feel very isolating. Participants commonly described experiences where they felt like they were intruding into a space where they were not wanted:

And there was a lot of angst in the room. It was like: ‘Don't come here and tell us what to do.’, type of thing… But the worst was being that sole person in a room of maybe 150 or 200 health professionals at that time, not… not being accepted.

P8

But sometimes, you know, you know, it was difficult. It was difficult to… to interrupt this meeting that clearly had, like, an established flow with people that have worked together for a very long time.

P12

The feeling of isolation was also sometimes exacerbated because the role of the HCR was not well understood or accepted by the public. One participant described their experience of isolation:

It is changing, but there is still a lot of stigma, is the word I would use, around what we do [patient safety advocacy] …It can be a very lonely place. People have to do a lot of work to not be the only person they know doing this type of thing.

P5

The ‘othering’ of HCRs was also underpinned and reinforced when systems did not accommodate them equitably (see more in Theme 2).

Conversely, the ‘otherness’ of HCRs was seen as their strength. Participants saw themselves as providing a unique outside perspective. Participants achieved a sense of acceptance when their contributions were recognised as expertise and sought out by others (see Theme 4). Having a differing perspective was often described as the purpose of HCRs:

You need to push for [change], because it's the people, it's the other people in that room who are going to talk about the budget and the organisational capacity, and they're not, they're not big dreamers in that sense.

P7

#### 3.2.2 Theme 2 – Effects of Personal and Structural Power

While some participants felt they were considered as equals in their groups, many described having less power than others, which contributed to feeling'othered’. For example, one relatively new HCR described:

It was really great in my first meeting, because it was such a high‐ranking committee. …. I guess it didn't bug me straight away that I wasn't given many opportunities to talk.

P7

The same participant continued to explain that eventually they were frustrated that their contributions were rarely sought.

Chairpersons, or others with power in these groups, were described as having a great influence over the involvement of HCRs. Feeling heard and respected by those with influence was how participants commonly experienced power being shared with them. This participant's description captured successful power sharing well:

[A good chairperson is] very inclusive and proactively inclusive, and I think that that has then had a huge impact on the rest of the group who actually see that respect and then experience the input that we bring.

P13

The influence participants had varied greatly between individuals, but also between the different opportunities they were involved in. The possibility for influence was often based on the perceived status or knowledge of HCR involvement and the culture of the group. According to one participant:

…that everyone's an equal when they're in a meeting and has equal say and equal weight, and it really depends on the attitude of the other people in the room, essentially. And attitude, not necessarily in terms of, you know, the personal behavioural attitude, but the attitude or mindset to, you know, [towards] dealing with consumers.

P1

Participants described their experiences of structural power inequities, including examples of systems‐factors that diminished or enhanced their opportunities for influence. Often participants felt they were too outnumbered to have HCR perspectives meaningfully acknowledged. Many of the opportunities that participants described were advisory, rather than vote‐casting, roles. One participant describes their voting role as culture‐changing in itself:

I've appreciated having that equal power with my colleagues, not above them, but equal power with them. Because I think it changes minds too.

P5

The formal nature of meetings could feel alien, as could the jargon. Procedures which were often simple for other members of groups, for example reimbursement for time and expenses and access to communication and documentation, could be complex and/or inequitable for HCRs. One participant described the positive impact of good communication:

You have other major services that do investigations incredibly well, and they're a joy to work with, and they want to share information with you, and they run them so well…

P13

Participants described how system barriers could make HCR involvement activities feel very tokenistic, prohibiting genuine influence. One HCR told of the impact of being involved at a later stage than other stakeholders:

But then when I understood that I hadn't been at the first two [codesign workshops]… It all started to make sense, why I feel like, so inadequate, like, why I can't, why can't I contribute? And I was desperately trying to, like, understand, but really I needed, like, needed someone to tell me, you know, what's happened before now, or ideally, you you're involved from the beginning.

P12

Addressing structural power imbalances was something many participants advocated for in their roles as HCRs.

#### 3.2.3 Theme 3 – Relational Aspects

Relational aspects were described by participants as underpinning their HCR experience. Interpersonal relationships were connected to power being shared or brokered and strongly shaped how HCRs perceived the quality of their experiences.

Both organised networks and informal connections between HCRs were important to participants for emotional support and to combat loneliness and feelings of otherness:

Oh, yeah, you have to be [relational]! Because you don't get paid for doing that kind of work, so you have to get hugs!

P4

Connections with other HCRs helped participants feel they were a part of a larger movement, as well as providing mentorship and reassurance. Participants also described that the friendships they made through their HCR involvement were key motivating factors:

I do [have friendships], like so with some consumers, they're really good with like crossing that emotional loop.

P10

Allyship was a key relational aspect described by participants. Allies could be HCRs or other supportive parties, like health consumer‐ or lived experience organisations, who would ‘have your back (P6)’. However, insider allies like clinicians, politicians and/or bureaucrats who recognise the value and expertise of consumers were also vital to achieving a common cause:

But it's not always just networks with other consumers. You often find you have allies on boards who are clinicians or bureaucrats, and they are wanting to achieve what you want to achieve as well, but they can't do it because of constraints that they have because of who they are employed by.

P2

Importantly, these insider allies also showed person‐to‐person expressions of respect and appreciation. One participant told of a very positive experience in forming an ally:

…And they're the Chief Medical Officer of [State 5] now. And [name removed] took me out for coffee one in those early days, and they said, I want to know, I want to hear your story…And I joke, it was the longest conversation with a doctor in my life.

P3

Accompanying this concept of allyship was collegiality and teamwork. Considering Theme 1, feeling like ‘the only consumer’, becoming a part of a team was particularly impactful. Participants described the joy and satisfaction that could be achieved when they reached a state of: ‘It's not us and them anymore’ (P13).

When people start asking you questions and really listen to the answers, and when you start to see progress being made in a line of inquiry, when you see people forgetting that you're a consumer representative and seeing that you're actually really interested in helping them out. So, it opens up a collegial feeling around the table, that's when you feel safe to say what you want to say.

P13

#### 3.2.4 Theme 4 – Becoming an Expert

As HCRs, participants described their role in a group as comprising multiple functions. These included representing voices that could not be ‘at the table’, providing an outsider perspective/holding to account, contributing lived experience and advocacy for justice and system improvement. One participant explained how they conceptualise one of their functions as providing a lay perspective, which challenges internal cultures in the health system:

I'm a professional pub test, right? It's my job to be, like: ‘You know, do you think that would fly like, out there in the world or in this population?’… And there's a lot of: ‘Oh, but this is normal’ from them and me saying: ‘okay, but should it be?’

P5

Participants often described in detail how they strategically used advocacy skills, knowledge, language and lived experience to gain respect and work towards a good outcome. According to one:

It's all about emotional buy‐in in bureaucratic system! Even though we might want to change it, it is so hard to change this if you don't be that strategic.

P10

Different qualities and skills were needed for effective HCR involvement and participants invested energy into developing their strategies. One participant described their take on effective HCR involvement

You look at things strategically, mesh that through with the actual on the ground, day to day, real life experience of being a carer… When you put that together, it's quite powerful… It's looking at the big picture whilst at the same time relaying the concerns of the day‐to‐day life experience of people living the health system or living with disease.’

P11

Participants also described their considerable effort to continuously engage and hear from a broad range of fellow consumers (service users) to be able to represent a range of experiences.

Being able to contribute a lived experience perspective was the strongest foundation of HCR expertise. With Theme 1 in mind, it was also often a unique expertise that was sought from the HCR as the ‘only consumer’ and valued by the group:

So what I'm finding is, I think people are embracing consumers now as part of the team, and realise that we do have a part to play, or some insights, maybe that's because we've had the lived experience.

P1

Lived experience and the associated story‐telling was used with care, purpose and skill. In system‐level spaces lived experience was used to remind the group that ultimately their purpose is the health and wellbeing of individuals:

I never shy away from sharing a personal story if I think it's relevant and it feels like the space in the meeting to do it, because I think it's sometimes the discussions around policy, you know, these high‐level decisions that are being made can forget or lose sight of the… the actual human experience…

P12

However, confidence in one's expertise was not a given. Participants described the journey toward gaining confidence in their contributions and expertise:

I was really quite scared and self‐conscious in the beginning…

P3

This journey took time, mentorship, and affirming HCR experiences. Participants often compared themselves to others in the room, who they perceived as experts in their own fields. They sometimes felt the need to prove to themselves and to others that they had a comparable level of value to contribute. One participant reflected:

I look back on the amount of training I've done, the amount of committees I've sat on, and the amount of times I've made a difference, and then I think: ‘I am on the same level as you. Just because I don't have a doctor's degree doesn't mean that I don't know what I'm talking about.’

P6

However, participants commonly discussed experiences where their expertise was not wanted or accepted. They described being dismissed, shut down and even bullied. Sometimes, there was no space or opportunity to contribute at all:

I'm really determined, and I have something to say, and I genuinely have experiences, and I'm trying to make that known to the organisation, and they actually just don't want to hear it. My things don't make it into the minutes. I sometimes get shut down by people.

P7

Often, there was a lack of understanding about the HCR role and a lack of understanding from other group members about what an HCR could add to a discussion. One participant summarises:

I call it the classic cycle of underestimation. So, when you first turn up new to something… it'll vary… but generally you'll get this sort of level of expectation that's very low about what you're going to add, how intelligent you're going to be, and how educated you're going to be, whatever. You know, people have all kinds of really infantilizing assumptions about consumer type people.

P5

This can mean that it fell to the participants themselves to advocate for the value of HCR involvement:

…then you're still back at that point of going: ‘now, this is what a consumer representative is’ and teach them, right from the word go, why your input is valuable, or what experience you have, and why you're around that same table with them… you spend a lot of time and effort trying to get included in the in the discussions and being taken seriously.

P13

Many, if they persisted in this type of environment, described being able to overcome these misunderstandings, as well as their own self‐doubts. The description of having to prove oneself to win support and approval from others in the group was common:

But I think you've… like in so many things… you have to earn your stripes. You have to demonstrate your sincerity, your honesty and your passion.

P8

#### 3.2.5 Theme 5 ‐ Finding Meaning in the Everyday to the Long‐Term

Throughout the interviews participants described finding meaning in everyday interactions as well as in big achievements. Overall, HCRs aimed to achieve meaningful systems change. However, they also derived meaning and even joy from successful teamwork, being part of the HCR movement, personal motivation, and even gestures of respect and acceptance.

Several participants described their motivations for being an HCR as two‐fold. One participant put it simply:

It's good for me and good for others!

P9

HCR experiences were sometimes described as ‘enjoyable’ (P4) when participants felt like they were a valued member of a productive team. Similarly, strong relationships as well as expressions of gratitude and respect from valued group members were seen as giving meaning to their role (refer to Theme 3).

However, the core purpose of HCR involvement was to make a difference. Participants had experienced significant health challenges or gaps in the system. Trying to address those issues gave meaning:

I mean, I might not be able to fix my son, but I could help make the system better for other people.

P9

Systemic change was seen as a ‘long game’ (P4) and participants described frustration about this, but also great satisfaction when they could see the impacts of their influence over time:

The actions that you do and that your colleagues do, kind of diffuse into the system, and then later on, you can see that the density of your social justice particles is slowly rising over time, if that makes sense. And so, when I first started this work, I was really sad, because I never… it took, it's taken six, 7 years to see that kind of change.

P4

Participants described wanting to achieve big change in the long run, but that small wins could keep them going:

But we're having sort of little wins along the way… so having little milestones like that, I think, make it rewarding, and fill my cup, so to speak.

P11

Sometimes meaning was derived from having access to influential people and the hope this gave them for significant change.

Some participants were also motivated to continue their efforts simply because they had hope that with the power of HCR advocacy the system will improve.

I believe that… in these roles, I can make a difference.

P12

Others felt that working towards better HCR engagement was often a purpose and meaningful outcome in itself:

So, I think building up an expectation that it was normal to have a consumer input was what I valued the most.

P2

## 4 Discussion

The interpretive phenomenological analysis of interview data uncovered five interconnected group experiential themes shaping HCRs’ experiences of system‐level involvement. Particularly prominent is the feeling of being the ‘only consumer’, which framed HCRs’ sense of distinctiveness and the isolation this can cause. The impact of power dynamics and relational aspects highlighted the critical role of systems factors, supportive relationships and allyship in shaping meaningful involvement. Important elements of HCR involvement described by participants were the journey of becoming an expert in their own eyes, as well as finding meaning and fulfilment in their roles.

### 4.1 Being ‘The Only Consumer’

Prominent across the HCR experience was the theme of being ‘the only consumer’, which shaped their place in decision‐making and carried unique expectations and challenges. This experience went beyond simply needing more HCRs in the room. It marked them as having a distinct perspective, which could lead to either marginalisation or acceptance, depending on group dynamics and attitudes. ‘Othering’ is a social phenomenon in which divisions between individuals and groups create an ‘us and them’ mentality [30]. In groups, ‘othering’ often leads to power imbalances, discrimination and prejudice towards the group that is othered [31]. In the traditionally hierarchical health care system, social identities within groups of different health care professionals impact group behaviour and collaboration [32]. At the system‐level, HCRs face challenges entering this group hierarchy because their social role shifts from being a patient to a decision‐making partner [33]. Wales et al. [19] conceptualise success at this key stage as the ‘practice of respect’. This study strongly echoed this finding, showing that whether the difference in perspective that HCRs bring ‘to the table’ was treated as unique expertise or as unwanted intrusion depended on the actions of other group members.

### 4.2 Effects of Personal and Structural Power and Relationships Aspects

Addressing power imbalances lies at the heart of the consumer movement [34]. In group settings, many other publications on HCR involvement have identified power imbalances between HCRs and other group members as barriers to true partnership [10, 18, 19, 20, 21, 22]. Adjacent to the concept of ‘othering’ described earlier, Jacob et al. [30] describe a concept of structural othering which develops between health professionals and patients because of the structural (rather than social or interpersonal) factors that influence interactions. Similarly, the present study highlights how system factors like language (jargon), remuneration and information access are experienced as structural power imbalances.

Interestingly, digital literacy or the use of technology did not arise as a concern among participants but has been described in other studies as a barrier to consumer and community involvement [35]. This may be due to selection bias as our participants were interviewed using a digital platform. Health informatics and digital technologies may increasingly offer solutions to some identified structural barriers, such as addressing information asymmetry through better designed shared digital workspaces or consumer‐facing knowledge repositories [35, 36]. However, it is important to note that HCRs are often working in systems that are not ‘theirs’. Structural barriers affect HCRs inequitably and should be recognised as disempowering, rather than simply inconveniencing [37].

The importance of allyship to broker power and support HCR engagement is similar to Happell et al.'s [38] findings in mental health lived experience advocacy. In addition, the crucial role of the chairperson echoes that of HCR experiences in patient safety investigations [21]. Relational factors for HCRs extend beyond professional allyship relationships to more personal ones. Referring back to the concept of ‘the practice of respect’ [19], this study expands the concept of allyship to show how being treated respectfully can extend to those in power showing genuine interest in their experiences and ideas.

### 4.3 Becoming an Expert and Finding Meaning

The expertise of HCRs is not routinely acknowledged, and this is keenly felt. It is a common experience that HCRs feel they must advocate for their own value [19, 21] as a part of their role. McPhee et al.'s study [33], tellingly titled ‘‘‘They don't really know why they're here’ mental health professionals’ perspectives of consumer representatives’’ explains ambiguity around HCR expertise and describes a culture which devalues lived experience and impedes meaningful HCR involvement. Similarly, Ehrlich, Lattery and Kendall [22] found that HCRs felt that if they did not affirm the efforts or ideas of health services staff their opinions often became unwanted. This study's findings may also be understood through the lens of epistemic injustice [39], whereby certain forms of knowledge are afforded less credibility than others because of existing power relations. Supporting this proposition, participants’ accounts suggest that lived‐experience knowledge was often not regarded as equivalent to professional or clinical expertise, requiring HCRs to continually establish their credibility before their contributions were recognised. This raises questions about how HCR role is perceived by others, given that HCRs themselves viewed their unique contribution as brining lived experience and perspectives that differ from those of clinicians and policymakers.

Taking the view that HCRs are experts in the phenomenon of their own involvement [19], this study begins to shed light on how HCRs view their role in health system‐level engagement. Participants described their expertise as a blend of lived experience, representation, and advocacy. They often described tailoring these three aspects of their expertise according to circumstance. Importantly, becoming an expert in one's own eyes is a journey that individual HCRs must undergo. However, the way their expertise is perceived by others is constantly negotiated depending on group dynamics. For many, especially novice HCRs, it could be difficult to maintain confidence if their expertise is not acknowledged by those who do have power in the system. Conversely, when the expertise of HCRs is recognised and sought, it is experienced as a sharing of power [40].

Motivations described in service‐level engagement [41] arose from positive healthcare experiences and a desire to ‘help the hospital’ deliver a service. Contrarily, this study's participants described their initial motivating factor as significant health challenges or the experience of service gaps. It is unclear whether this is because there is a true difference between the system‐level and service‐level involvement or caused by differences in study methodology. Tellingly, however, even when there is high confidence in the service provider, there are still calls to improve HCR involvement to make it more meaningful [41]. In this study, as in others [21, 22], HCR involvement was described as a rewarding, albeit difficult role. This study adds insights into how motivation for continued involvement need to be reinforced by meaningful experiences and achievements.

### 4.4 Policy and Practice Implications

These results provide a key insight into the lived experience of being an HCR in system‐level spaces. The central objective of the research is to elevate the insights and opinions of HCRs as experts in their own involvement. In doing so, the study contributes to the academic discourse on the phenomenon of HCR involvement by integrating HCR voices into its narrative. It challenges assumptions of expertise that may traditionally be awarded to health care professionals, who are often regarded as default experts in the field of health [19, 20, 42]. These assumptions extend to research on HCR involvement, where it is rare to find research on the topic which does not also include service providers as participants, or where HCR experiences are a small part of a larger study.

The focus of this study is to highlight the lived experience of being an HCR. It does not necessarily aim to recommend how to best involve consumers; for this, there are many resources, developed by government departments as well as consumer engagement organisations in Australia and internationally. Additionally, a Cochrane review by Merner et al. [18] provides co‐produced recommendations for best principles in partnering with consumers. Instead, this study's findings should prompt those who wish to engage HCRs to consider their own experiences and preconceptions around their position in groups, power dynamics, relational aspects, becoming an expert and finding meaning in their involvement. These findings demonstrate the impact of different practices on individuals that must be considered when working away from tokenism and towards consumer leadership. Internationally, various structures and frameworks have been developed to support consumer involvement from the service level through to research and system‐level policy development. These include Canadian Institutes of Health Research's Citizen Engagement Framework [43], the United Kingdom's National Institute for Health and Care Excellence's Patient and Public Involvement Policy [44] and the upcoming Australian Department of Health, Disability and Aging's National Consumer Engagement Strategy for Health and Wellbeing [45]. Applying this understanding alongside best‐practice frameworks may help to overcome entrenched barriers to meaningful partnership.

Importantly, the role ambiguity of HCRs is a key opportunity for improvement in practice, and also an area for further research. In health system‐level spaces, role ambiguity [6], as well as ‘conflicting notions of valid knowledge’ [23] are key barriers to partnership with HCRs. Conceptualising HCR participation at the system and policy‐setting level is particularly unclear, which may be partly due to semantics [13]. Here, the very concept of ‘representation’ is flawed. Indeed, if HCRs are merely seen as representatives of broader populations, it could be damaging to the movement when they fail this impossible task [14, 46]. Sandman et al. [34] assert the need to rethink the traditional role of HCRs as ‘representatives’ at this level and argue for the concept of a ‘consumer consultant’ instead. This study similarly posits the need for a more multi‐faceted understanding of the role in which HCRs see themselves in system‐level spaces. Considering consumer sentiment uncovered in this study, the term ‘consumer expert’ could be more apt.

### 4.5 Strengths and Limitations

A strength of this study is its broad focus on HCR involvement at the system‐level, rather than focusing on one specific group or service. It also centres on the rich descriptions of the lived experiences of HCRs themselves, rather than relying on data about HCR involvement from health service staff. Another strength of this study is the diversity of its participants and their HCR experiences (see Section 2.2).

A limitation is that, while participants were recruited for their experience in system‐level HCR involvement, they also often had other HCR roles, which were at the health service‐level, for example on hospital committees. Although prompted to consider their system‐level roles in the interviews, discussions naturally included their experiences more broadly.

Researcher HF works for a health consumer organisation, has expertise in HCR involvement and personal lived experience as an HCR. This dual role as researcher and HCR insider may have shaped both rapport and disclosure. While this position supported trust and shared understanding, it may also have influenced which experiences participants chose to emphasise, including positive accounts of allyship or more critical accounts of power and exclusion. The reflexive, hermeneutic approach [27] and research team discussions were used to consider how HF's positionality may have shaped the interview interaction, participant disclosure and interpretation of the data.

## 5 Conclusion

HCRs are passionate about their involvement, and the movement has undergone significant changes in recent decades. However, there are further opportunities to improve the quality of HCR involvement. Recommendations for best practice HCR involvement continue to evolve. This study contributes a nuanced understanding of HCRs’ experiences, which helps to contextualise the need for and use of engagement strategies. These insights are particularly important at the relatively underexplored health system‐level. This research intends to bring to life the difficulties and triumphs of HCRs and provides health professionals, staff, researchers and policy makers with a clearer understanding of how their actions shape HCR involvement. This evidence can be used to build true partnerships with HCRs and reduce tokenistic involvement, thereby increasing the substantive impact of HCRs at the health‐system level.

## Author Contributions

**Hannah Fielder:** writing – original draft, methodology, conceptualisation, investigation, formal analysis, writing – review and editing. **Rebecca Kelly:** supervision, methodology, conceptualisation, writing – review and editing, validation. **Phoebe Griffin:** supervision, writing – review and editing, resources, validation.

## Ethics Statement

This study has been approved by the University of Tasmania Human Research Ethics Committee (H0031018).

## Conflicts of Interest

The authors declare no conflicts of interest.

## Supporting information


Supporting File

## Data Availability

All data are confidential due to ethical considerations.

## References

[1] World Health Organization , Declaration of Alma‐Ata: International Conference on Primary Health Care, Alma‐Ata, USSR, Alma‐Ata, USSR1978.

[2] K. Josje , P. Flora , A. Nada , S. Linda , B. Hester van de , and P. Anne Margriet , “7 Working in Partnership: Service User Involvement in Healthcare Regulation,” supplement, BMJ Open Quality 14, no. Suppl 3 (2025): A5–A6.

[3] Australian Commission on Safety and Quality in Health Care (ACSQHS) , The NSQHS Standards ‐ Partnering With Consumers Standard: ACSQHS; 2024, ttps://www.safetyandquality.gov.au/standards/nsqhs-standards/partnering-consumers-standard.

[4] B. Scholz , “We Have to Set the Bar Higher: Towards Consumer Leadership, Beyond Engagement or Involvement,” Australian Health Review 46, no. 4 (2022): 509–512. 10.1071/AH22022 35508415

[5] J. A. Wolf , “Four Commitments for the Future of Healthcare: Reflecting on a Decade of Patient Experience Journal,” Patient Experience Journal 10, no. 3 (2023): 1–5.

[6] H. J. Westerink , T. Oirbans , M. M. Garvelink , et al., “Barriers and Facilitators of Meaningful Patient Participation at the Collective Level in Healthcare Organizations: A Systematic Review,” Health Policy 138 (2023): 104946. 10.1016/j.healthpol.2023.104946 38000333

[7] International Association of Public Participation (IAP2) , IAP2 Spectrum of Public Participation: IAP2 International; 2018, https://iap2.org.au/resources/spectrum/.

[8] L. A. Baumann , A. K. Reinhold , and A. L. Brütt , “Public and Patient Involvement in Health Policy Decision‐Making on the Health System Level ‐ A Scoping Review,” Health Policy 126, no. 10 (2022): 1023–1038. 10.1016/j.healthpol.2022.07.007 35918211

[9] K. W. Billing , Strengthening Consumer Engagement ‐ Summary Report (Commission on Excellence and Innovation in Health, 2022).

[10] A. E. Hall , J. Bryant , R. W. Sanson‐Fisher , E. A. Fradgley , A. M. Proietto , and I. Roos , “Consumer Input Into Health Care: Time for a New Active and Comprehensive Model of Consumer Involvement,” Health Expectations 21, no. 4 (2018): 707–713. 10.1111/hex.12665 PMC6117488 29512248

[11] T. Andreassen , “Service User Involvement and Repositioning of Healthcare Professionals: A Framework for Examining Implications of Different Forms of Involvement,” Nordic Welfare Research 3 (2018): 58–69.

[12] L. Horvat , Partnering in Healthcare for Better Care and Outcomes (Safer Care Victoria, State Government of Victoria, 2019).

[13] T. Rooke and A. Oudshoorn , “Patient Engagement in the Nonclinical Setting: A Concept Analysis,” Nursing Forum 55, no. 3 (2020): 497–504. 10.1111/nuf.12455 32324910

[14] B. Scholz , L. Kirk , T. Warner , L. O'Brien , Z. Kecskes , and I. Mitchell , “From a Single Voice to Diversity: Reframing ‘Representation’ in Patient Engagement,” Qualitative Health Research 34 (2024): 1007–1018. 10.1177/10497323231221674 PMC11487870 38229426

[15] A. Conklin , Z. Morris , and E. Nolte , “What Is the Evidence Base for Public Involvement in Health‐Care Policy?: Results of a Systematic Scoping Review,” Health Expectations 18, no. 2 (2015): 153–165. 10.1111/hex.12038 PMC5060770 23252574

[16] A. B. Sandvin Olsson , A. Strøm , M. Haaland‐Øverby , K. Fredriksen , and U. Stenberg , “How Can We Describe Impact of Adult Patient Participation in Health‐Service Development? A Scoping Review,” Patient Education and Counseling 103, no. 8 (2020): 1453–1466. 10.1016/j.pec.2020.02.028 32098746

[17] C. P. Pagatpatan and P. R. Ward , “Understanding the Factors That Make Public Participation Effective in Health Policy and Planning: A Realist Synthesis,” Australian Journal of Primary Health 23, no. 6 (2017): 516–530. 10.1071/PY16129 28641704

[18] B. Merner , L. Schonfeld , A. Virgona , et al., “Consumers’ and Health Providers’ Views and Perceptions of Partnering to Improve Health Services Design, Delivery and Evaluation: A Co‐Produced Qualitative Evidence Synthesis,” Cochrane Database of Systemtic Reviews 3, no. 3 (2023): Cd013274. 10.1002/14651858.CD013274.pub2 PMC10065807 36917094

[19] C. R. Wales , J. A. Lababedi , A. Coles , P. Lee , and E. Clarke , “Consumer Representative Experiences of Partnership With Health Workers in Australia,” Patient Experience Journal 8, no. 3 (2021): 64–78.

[20] B. Scholz , J. Bocking , P. Hedt , V. N. Lu , and B. Happell , “‘Not in the Room, but the Doctors Were’: An Australian Story‐Completion Study About Consumer Representation,” Health Promotion International 35, no. 4 (2019): 752–761. 10.1093/heapro/daz070 31325360

[21] P. D. Hibbert , Y. Yu , C. J. Molloy , et al., “Perceptions and Experiences of Consumer Representatives on Patient Safety Investigation Teams: A Qualitative Analysis,” Health Expectations 28, no. 3 (2025): e70281. 10.1111/hex.70281 PMC12042694 40304616

[22] C. Ehrlich , M. Slattery , and E. Kendall , “Consumer Engagement in Health Services in Queensland, Australia: A Qualitative Study About Perspectives of Engaged Consumers,” Health & Social Care in the Community 28, no. 6 (2020): 2290–2298. 10.1111/hsc.13050 32511875

[23] M. B. Steffensen , C. L. Matzen , and S. Wadmann , “Patient Participation in Priority Setting: Co‐Existing Participant Roles,” Social Science & Medicine (1982) 294 (2022): 114713. 10.1016/j.socscimed.2022.114713 35032747

[24] D. Ezzy , Qualitative Analysis (Taylor & Francis Group, 2002).

[25] J. W. Creswell and C. N. Poth , *Qualitative Inquiry & Research Design: Choosing Among Five Approaches*. 4th ed. (SAGE Publications, 2018).

[26] P. Liamputtong , K. Anderson , and T. Bondas , Research Methods in Health (Oxford University Press, 2016).

[27] B. E. Neubauer , C. T. Witkop , and L. Varpio , “How Phenomenology Can Help Us Learn From the Experiences of Others,” Perspectives on Medical Education 8, no. 2 (2019): 90–97. 10.1007/s40037-019-0509-2 PMC6468135 30953335

[28] J. L. Johnson , D. Adkins , and S. Chauvin , “A Review of the Quality Indicators of Rigor in Qualitative Research,” American Journal of Pharmaceutical Education 84, no. 1 (2020): 7120. 10.5688/ajpe7120 PMC7055404 32292186

[29] D. L. Kahn , *Hermeneutic Phenomenological Research: A Practical Guide for Nurse Researchers. 2000 2024/05/23* (SAGE Publications, Inc., 2012). https://sk.sagepub.com/books/hermeneutic-phenomenological-research.

[30] J. D. Jacob , M. Gagnon , A. Perron , and M. K. Canales , “Revisiting the Concept of Othering: A Structural Analysis,” Advances in Nursing Science 44, no. 4 (2021): 280–290. 10.1097/ANS.0000000000000353 33657021

[31] D. Bhugra and A. Ventriglio , “Others, Othering, Otherism and Social Psychiatry,” International Journal of Social Psychiatry 70, no. 5 (2024): 837–838. 10.1177/00207640241269086 39082105

[32] S. A. Kreindler , D. A. Dowd , N. Dana Star , and T. Gottschalk , “Silos and Social Identity: The Social Identity Approach as a Framework for Understanding and Overcoming Divisions in Health Care,” Milbank Quarterly 90, no. 2 (2012): 347–374. 10.1111/j.1468-0009.2012.00666.x PMC3460209 22709391

[33] J. McPhee , T. Warner , T. Cruwys , B. Happell , and B. Scholz , “They Don't Really Know Why They're Here’ Mental Health Professionals’ Perspectives of Consumer Representatives,” International Journal of Mental Health Nursing 32, no. 3 (2023): 819–828. 10.1111/inm.13124 36727283

[34] L. Sandman , B. Hofmann , and G. Bognar , “Rethinking Patient Involvement in Healthcare Priority Setting,” Bioethics 34, no. 4 (2020): 403–411. 10.1111/bioe.12730 32333687

[35] K. Kiani , V. Phillips Ingvason Nguyễn , E. Siira , C. Wallengren , A. Wolf , and I. Björkman , “Tensions in Patient Involvement and Engagement in Health Research and Development: A Qualitative Interview Study With Key Actors,” Research Involvement and Engagement 12 (2026): 87, 10.1186/s40900-026-00915-3. PMC13255309 42277976

[36] A. H. Ng , S. Reeder , A. Jones , et al., “Consumer and Community Involvement: Implementation Research for Impact (Cciri)–Implementing Evidence‐Based Patient and Public Involvement Across Health and Medical Research in Australia–A Mixed Methods Protocol,” Health Research Policy and Systems 23, no. 1 (2025): 25. 10.1186/s12961-025-01293-0 PMC11849185 39994745

[37] M. E. Snow , “Patient Engagement in Healthcare Planning and Evaluation: A Call for Social Justice,” supplement, International Journal of Health Planning and Management 37, no. S1 (2022): 20–31. 10.1002/hpm.3509 35643909

[38] B. Happell and B. Scholz , “Doing What We Can, but Knowing Our Place: Being an Ally to Promote Consumer Leadership in Mental Health,” International Journal of Mental Health Nursing 27, no. 1 (2018): 440–447. 10.1111/inm.12404 29171920

[39] M. Fricker , Epistemic Injustice: Power and the Ethics of Knowing (Oxford University Press, 2007).

[40] B. Scholz , J. Bocking , and B. Happell , “Improving Exchange With Consumers Within Mental Health Organizations: Recognizing Mental Ill Health Experience as a ‘Sneaky, Special Degree’,” International Journal of Mental Health Nursing 27 (2018): 227–235. 10.1111/inm.12312 28145617

[41] C. Adams and A. J. Brown , “Partnering With Healthcare: The Experiences of Consumer Representatives,” Patient Experience Journal 10, no. 1 (2023): 4–9.

[42] J. Ocloo , S. Garfield , S. Dawson , and B. Dean Franklin , “Exploring the Theory, Barriers and Enablers for Patient and Public Involvement Across Health, Social Care and Patient Safety: A Protocol for a Systematic Review of Reviews,” BMJ Open 7, no. 10 (2017): e018426. 10.1136/bmjopen-2017-018426 PMC5665219 29070642

[43] Canadian Institutes of Health Research , Citizen Engagement Framework. Ottawa: CIHR; 2010, https://cihr-irsc.gc.ca/e/41270.html.

[44] National Institute for Health and Care Excellence , Patient and Public Involvement Policy (NICE, 2013).

[45] Australian Department of Health and Aged Care , Draft National Consumer Engagement Strategy for Health and Wellbeing (Australian Government, 2023).

[46] B. Scholz , S. J. Stewart , J. Bocking , and B. Happell , “Rhetoric of Representation: The Disempowerment and Empowerment of Consumer Leaders,” Health Promotion International 34, no. 1 (2019): 166–174. 10.1093/heapro/dax070 29036568

