# Supplementary material for: Being ‘The Only Consumer’ – Exploring the Lived Experiences of Health Consumer Representatives in Australian Health System Involvement
Source: Health Expect. 2026 Oct 5;29(5):e70901. doi: 10.1111/hex.70901 (PMC13636653; doi:10.1111/hex.70901)
Supplement: Supplementary file 1 [file HEX-29-e70901-s001.docx]

## Appendix 1 – Semi-structured interview guiding questions

Rapport/ Background:

1. What types of consumer representation activities you have recently been involved in?
a. What is your role?
b. Who else is on the group?
c. Have you had a lot of experience doing consumer representation?
d. How do you explain your role to others?
e. Is the role current/ongoing or has it already happened?

Activating narrative construction:

2. Can you tell me about some of the experiences you have had in public-health decision-making?

Gauging priorities:

3. What are some of the most meaningful experiences you have had in these activities?

Broadening the scope:

4. Tell me of a time that was particularly successful/unsuccessful (opposite to what the questions 3 leans towards)

Filling in detail:

5. Pick a normal meeting/activity and tell me what happens (describe).
6. How do these experiences feel?
